# Identifying key factors associated with subscapularis tendon tears and developing a risk prediction model to assist diagnosis

**DOI:** 10.1186/s12891-022-05351-4

**Published:** 2022-04-27

**Authors:** Wennan Xu, Fei Wang, Qingyun Xue

**Affiliations:** 1 Orthopaedics Department, Beijing Hospital, National Center of Gerontology, Institute of Geriatric Medicine, Chinese Academy of Medical Sciences, NO.1 DaHua Road, Dong Dan, Beijing, 100730 PR China; 2 Graduate School of Peking Union Medical College, NO.1 DaHua Road, Dong Dan, Beijing, 100730 PR China

**Keywords:** Subscapularis, Nomogram, Tear, Prediction

## Abstract

**Background:**

There are still some challenges in diagnosing subscapularis (SSC) tendon tears as accurately as posterosuperior rotator cuff tears on MRI. The omission of SSC tendon tear can lead to muscle atrophy, fatty infiltration, and increased tearing accompanied by aggravated shoulder pain and loss of function. An effective non-invasive evaluation tool will be beneficial to early identification and intervention. The study aims to identify sensitive predictors associated with SSC tendon tears and develop a risk prediction model to assist in diagnosis.

**Methods:**

Data on 660 patients who received shoulder arthroscopic surgery with preoperative shoulder MRI were collected retrospectively. Of these, patients with SSC tendon tears were defined as the SSC tear group, and patients with intact SSC tendon were enrolled in the non-SSC tear group. Logistic regression analysis was used to identify the key predictors of SSC tendon tears which were then incorporated into the nomogram.

**Results:**

Among 22 candidate factors, five independent factors including coracohumeral distance (CHD, oblique sagittal plane) (OR, 0.75; 95%CI, [0.67–0.84]), fluid accumulation (Y-face) (OR, 2.29; 95%CI, [1.20–4.38]), long head of biceps tendon (LHB) dislocation/subluxation (OR, 3.62; 95%CI, [1.96–6.68]), number of posterosuperior (PS) rotator cuff tears (OR, 5.36; 95%CI, [3.12–9.22]), and MRI diagnosis (based on direct signs) (OR, 1.88; 95%CI, [1.06–3.32]) were identified as key predictors associated with SSC tendon tears. Incorporating these predictors, the nomogram achieved a good C index with a good agreement on the risk estimation of calibration plots. Higher total points of the nomogram were associated with a greater risk of SSC tendon tears.

**Conclusion:**

When evaluating the severity of SSC tendon injury, the combination of reliable predictors can improve the sensitivity and diagnostic performance of MRI. This model provides an individualized probability of risk prediction, which is convenient for clinicians to identify patients at high risk for SSC tendon tears to avoid missed diagnosis.

## Introduction

Rotator cuff injury is a common cause of shoulder pain and dysfunction. As the largest and most powerful tendon in the rotator cuff, the subscapularis (SSC) tendon is an important component in maintaining transverse force couple balance and plays an important role in the stability of the glenohumeral joint and internal rotation [1]. Approximately 12–50% of patients were reported to have SSC tendon tears undergoing arthroscopy [2–4]. MRI is a powerful non-invasive tool for diagnosing rotator cuff injuries. Although the sensitivity of MRI in the diagnosis of supraspinatus (SSP) and infraspinatus (ISP) tendon tears is more than 90%, it has been challenging to find MRI as an effective diagnostic tool for SSC [5–7]. Furukawa R et al. found that the sensitivity of 3.0-T magnetic resonance imaging (MRI) in diagnosing SSC tendon tears was 57.9% and 60.5% in the axial and oblique sagittal plane, respectively [8]. When the field strength was reduced to 1.5-T, the diagnostic sensitivity of conventional MRI for SSC tendon tears was only 45.3% [9]. A systematic review and meta-analysis demonstrated that the overall sensitivity of MRI in the diagnosis of SSC tendon tears was 68% [10]. However, more than half of the studies included in this review used magnetic resonance arthrography (MRA) as a diagnostic tool, which would improve its sensitivity.

The size and thickness of SSC tendon tears directly affected the diagnostic performance of MRI, the smaller the tear size, the lower the accuracy of diagnosis [11, 12]. Adams et al. found that SSC tendon tears that extended at least half the cephalad-to-caudal distance were more easily identified on high-field MRI [13]. For tears involving the superior one-third of the tendon insertion, the sensitivity of MRI was significantly reduced [11]. Based on a large number of cadaver specimens, Yoo and Rhee et al. divided the footprint of the subscapular tendon into four regions (facet 1 to facet 4) [14]. They concluded that SSC tendon tears usually started in the upper part of the lesser tuberosity insertion, and most occurred in the upper 1/3 region of the insertion, which is basically equivalent to the first facet (facet 1). Yoo and Rhee et al. classified SSC tendon tears into 5 types (type I to type V) according to the shoulder arthroscopy of 821 patients. Among those SSC tendon tears, less than or equal to 80% were facet 1 tears (upper 1/3 footprint), with major types of fraying and partial-thickness tears (type I/IIa/IIb). The characteristic of small partial tears in the upper 1/3 footprint as the main tearing type reduced the overall sensitivity and accuracy of MRI in SSC tendon tears.

Although a good clinical outcome was obtained after arthroscopic repair of SSC tendon tears [15–17], the current sensitivity of MRI in SSC tendon tears was not ideal. The preoperative omission of SSC tendon tears could result in long-term pain and dysfunction of the shoulder, accompanied by progression of muscle atrophy, fatty infiltration, and increased tearing [18]. Yoon TH et al. found that SSC tendon tears combined with advanced fatty infiltration had a high retear rate of 78.6% despite clinical improvement through surgery [19]. Among arthroscopic revision rotator cuff repairs, 43.1% had neglected SSC tears, and fatty infiltration of these initially neglected SSC tendons showed further progression at the time of revision [20]. When the SSC tendon tears extended more than the upper 1/3 of the tendon, the failure rate of massive posterosuperior rotator cuff tear repair was significantly increased [21, 22]. The higher incidence, low diagnostic sensitivity, participation of important functions, and clinical harm of missed diagnosis indicated that it was very important to improve the early recognition of SSC tendon tears.

Clinicians often evaluated the severity of SSC tendon injuries on MRI according to changes in tendon morphology and signal, but there were still some limitations [7, 12, 23]. A reliable risk estimation tool is helpful for the screening and early identification of patients with SSC tendon tears. With further investigation of SSC tendon injury, studies have found that subcoracoid impingement and coracoid morphological variation might play an important role in the pathological injury of SSC tendon [24]. In addition, scholars suggested that some indirect imaging signs might be associated with SSC tendon tears [25–27]. These suggested that finding reliable predictors highly associated with SSC tendon tears might improve the diagnostic sensitivity and accuracy of this disease.

Based on these reasons, we hope to identify and screen out reliable predictors highly associated with SSC tendon tears and develop a clinical prediction model to convert this speculative experience into scientific risk estimation to assist the diagnosis. Of all the available models, a nomogram can provide an evidence-based, individualized, and highly accurate risk estimation. Besides, the nomogram is easy to use and can facilitate management-related decision-making. This study hypothesized that this novel risk prediction model could provide superior diagnostic performance in predicting SSC tendon tears.

## Method

### Patients

This case-control study was approved by our institutional review board (2021BJYYEC-225-01). The requirement for patient consent was waived by the review board because of the retrospective nature of the study. We retrospectively reviewed 660 patients who received shoulder arthroscopic surgery between January 2016 and October 2021. Among them, 460 patients who underwent shoulder arthroscopic surgery from January 2017 to December 2020 were enrolled in the training cohort. A total of 200 patients with shoulder arthroscopic surgery from January 2021 to October 2021 and from January 2016 to December 2016 were entered into the validation cohort. Patients were divided into a training cohort (*n* = 460) and a validation cohort (*n* = 200) for development and external validation of the model, respectively. The inclusion criteria were as follows: (1) underwent primary arthroscopic shoulder surgery, (2) preoperative shoulder MRI was completed in our hospital with complete imaging data, and (3) all surgeries were performed by an experienced doctor specializing in shoulder arthroscopy. Patients combined with fractures, tumors, immunologically related diseases, rheumatic immune diseases, and revision surgery of the shoulder were excluded.

Characteristics of patients included age, gender, cause of injury, and injury side (i.e., left or right side). According to the classification of Lafosse [28], we evaluated the severity of SSC tendon tears under arthroscopy. Yoo and Rhee et al. divided the SSC tendon footprint into 4 distinct facets (facet 1 ~ 4) through a cadaveric observational study [14]. They provided the facet’s dimensions and surface area, which is convenient for arthroscopic measurement. We measured SSC tear size during the arthroscopy by a numeric probe with a scale of 1 mm to describe the classification (Lafosse I ~ V). Patients with arthroscopically determined SSC tendon tears were included in the SSC tear group, while others with intact SSC tendons were enrolled in the non-SSC tear group. A detailed description of characteristics was presented in Table 1.

**Table 1:** Characteristics and result of univariate logistic analysis

Variable	Training cohort		OR (95%CI)	*P* Value	Validation cohort	
	SSC tear (*n* = 184)	non-SSC tear (*n* = 276)			SSC tear (*n* = 86)	non-SSC tear (*n* = 114)
Age, year	62.55 ± 9.03	59.76 ± 9.42	1.03 (1.01–1.06)	0.002	63.12 ± 8.82	60.48 ± 9.13
Gender						
male	58	92	0.92 (0.62–1.37)	0.685	27	39
female	126	184			59	75
Cause						
degenerative	96	137	0.90 (0.62–1.31)	0.594	44	50
traumatic	88	139			42	64
Injured side						
right	112	155	1.21 (0.83–1.78)	0.316	53	70
left	72	121			33	44
CHD (axial), mm	8.04 ± 2.25	9.28 ± 2.46	0.79 (0.72–0.86)	< 0.001	7.88 ± 2.11	9.61 ± 2.44
CHD (oblique sagittal), mm	8.17 ± 2.08	9.73 ± 2.60	0.75 (0.68–0.82)	< 0.001	8.09 ± 2.02	9.94 ± 2.61
CO, mm	15.96 ± 3.84	15.84 ± 3.94	1.01 (0.96–1.06)	0.743	11.10 ± 3.92	15.45 ± 4.47
CHI	0.33 ± 0.08	0.52 ± 3.14	2.52 (0.22–28.94)	0.457	0.33 ± 0.08	0.31 ± 0.09
Fluid accumulation (En-face)						
yes	154	191	2.28 (1.43–3.65)	0.001	73	71
no	30	85			13	43
Fluid area ratio (En-face), ratio>0.5						
yes	30	12	4.29 (2.13–8.62)	< 0.001	14	5
no	154	264			72	109
Fluid accumulation (Y-face)						
yes	58	23	5.06 (2.99–8.59)	< 0.001	28	8
no	126	253			58	106
Fluid accumulation (Coronal)						
yes	122	140	1.91 (1.30–2.81)	0.001	60	43
no	62	136			26	71
Atrophy (En-face)						
grade I	132	256	5.04 (2.89–8.80)	< 0.001	25	9
grade II/III	52	20			61	105
Atrophy (Y-face)						
grade I	108	218	2.65 (1.75–4.00)	< 0.001	37	25
grade II/III	76	58			49	89
Subcoracoid cyst						
yes	106	73	3.78 (2.54–5.62)	< 0.001	53	27
no	78	203			33	87
Lesser tuberosity cyst, number ≥ 1						
yes	62	70	1.47 (0.98–2.21)	0.065	29	39
no	122	206			57	75
Lesser tuberosity cyst, diameter ≥ 5 (mm)						
yes	18	13	2.19 (1.05–4.60)	0.037	9	7
no	166	263			77	107
LHB dislocation/subluxation						
yes	70	24	6.45 (3.86–10.78)	< 0.001	33	16
no	114	252			53	98
Patte						
normal/grade I	120	246	4.37 (2.69–7.11)	< 0.001	31	4
grade II/III	64	30			55	110
Classification						
non full-thickness tear	88	219	4.19 (2.78–6.32)	< 0.001	38	95
full-thickness tear	96	57			48	19
Number of PS rotator cuff tears						
≤ 1	94	248	8.48 (5.22–13.79)	< 0.001	42	105
≥ 2	90	28			44	9
MRI diagnosis (direct signs)						
SSC tear	56	43	2.37 (1.51–3.73)	< 0.001	25	17
non-SSC tear	128	233			61	97

*LHB* Long head of biceps tendon, *SSC* Subscapularis, *CHD* Coracohumeral distance, *CO* Coracoid overlap, *CHI* Coracohumeral index, *OR* Odds ratio

All patients who underwent shoulder arthroscopic surgery were placed in the beach chair position with general anesthesia, and all of the procedures were performed by two comparably senior shoulder surgeons. The main surgical procedures include (1) arthroscopic exploration and debridement, (2) subacromial decompression, (3) adhesion release, (4) rotator cuff repair, (5) fixation of the labrum, (6) tenotomy or tenodesis of the long head of the biceps and (7) debridement of calcific tendinitis. If necessary, all patients were instructed to use a shoulder abduction brace immediately with identical rehabilitation protocol postoperatively. Rehabilitation training was conducted under the guidance of professional rehabilitation physicians.

### Imaging characteristics

All patients enrolled in the study received the identical imaging protocol. T1-weighted, T2-weighted fast-spin-echo, and fat-suppressed gradient echo and proton density-weighted (PDW) images (FOV = 220 mm; thickness = 4/5 mm) were performed underwent 3.0-T MRI scanner (Siemens Medical Systems) with the arm in a neutral position. None of the patients received magnetic resonance arthrography (MRA). According to previous reports [24, 26, 29–32], clinical experience, and clinical importance, seventeen imaging features were measured for further evaluation by two trained orthopedic doctors (LS and FW). The consensus was reached after deliberation and the mean value of variables was obtained with twice repeat measurements.

**Fig. 1:**
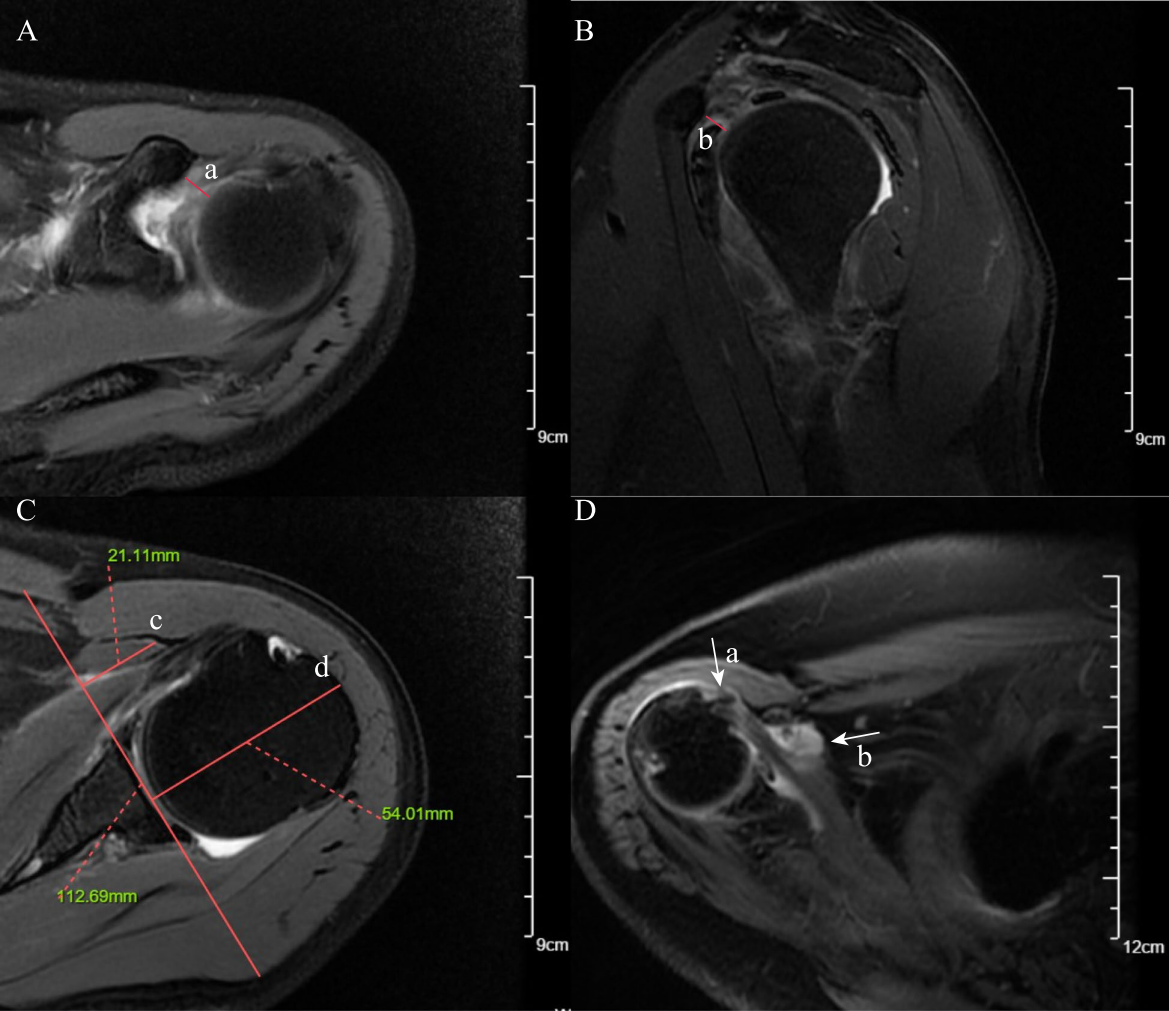
Fat-suppressed T2-weighted MRI images of coracohumeral distance (CHD), coracoid overlap (CO), coracohumeral index (CHI), LHB dislocation/subluxation and subcoracoid cyst. **A** CHD measured on axial plane (red solid line a). **B** CHD measured on oblique sagittal plane (red solid line b). **C** CO (red solid line c), CHI (red solid line c/ red solid line d, namely 21.11 mm/54.01 mm). **D** Subcoracoid cyst on axial plane (white arrow b). Dislocation/subluxation of LHB on axial plane (white arrow a)

**Fig. 2:**
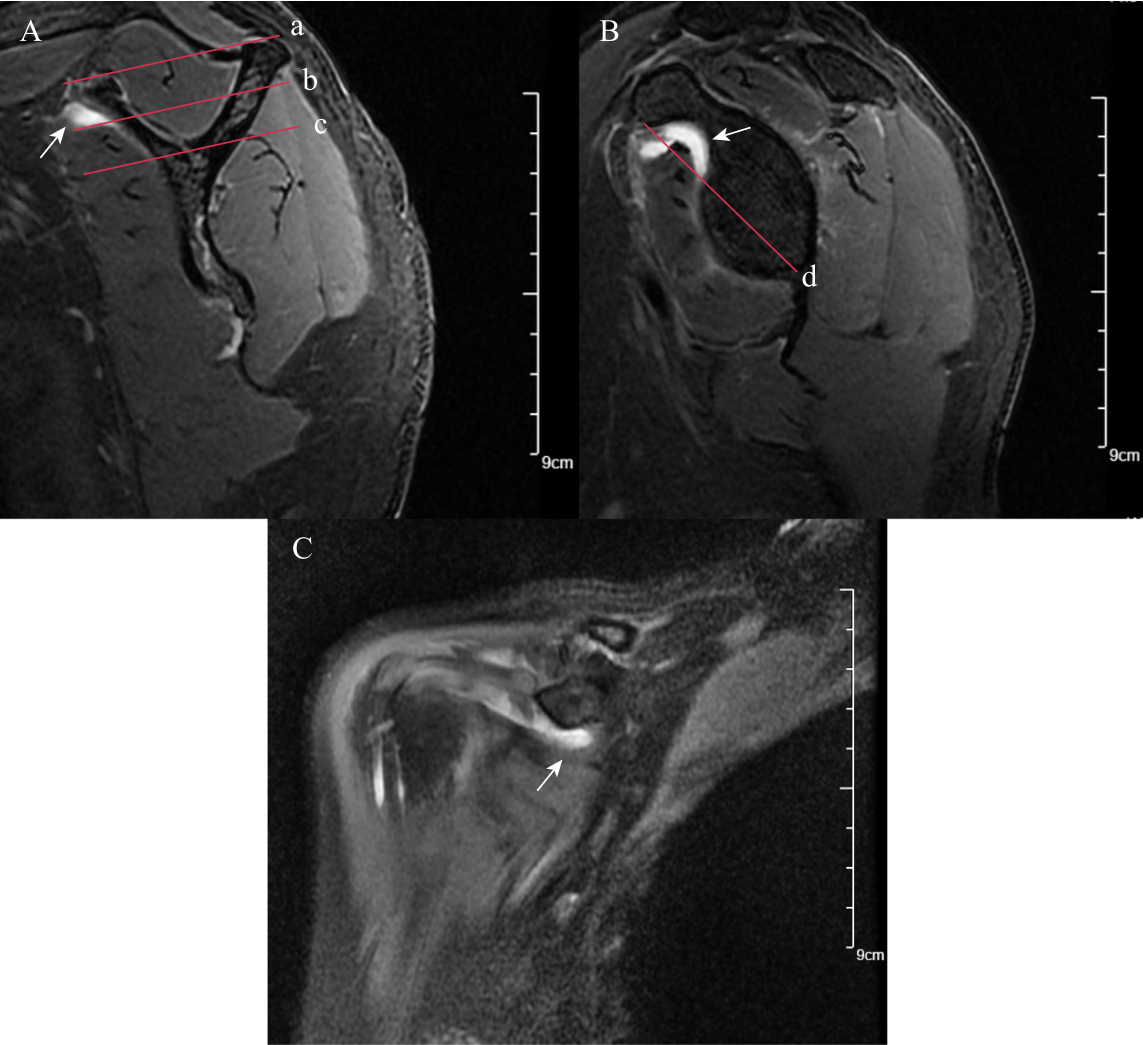
MRI images of subscapular muscle atrophy, fluid accumulation, and fluid area ratio (en-face). **A** SSC muscle atrophy classification on Y- face plane. Parallel line intercepting supraspinatus fossa opening (red solid line a), and parallel line (red solid line b) bisecting the perpendicular distance between line (red solid line a) and line (red solid line c). Based on these lines, SSC position was classified. Between line (a) and (b) was classified as grade (I), between line (b) and (c) was classified as grade (II), below line (c) was classified as grade (III). Fluid accumulation in the Y-face plane (white arrow). **B** En-face plane showing base to-tip line (BTL): the inferior pole of glenoid to coracoid tip on en-face (red solid line d). Using BTL, muscle atrophy of SSC is classified as grade I (tendon and muscle exist above the BTL), grade II (only tendon exist above the BTL), and grade III (tendon and muscle exist below the BTL). The fluid area ratio was the ratio of the effusion area to the area surrounded by the coracoid process, glenoid, and BTL in the en-face. Fluid accumulation in the en-face plane (white arrow). **C** Fluid accumulation in the coronal plane (white arrow)

Coracohumeral distance (CHD) was measured from the humeral cortex to the coracoid process cortex [24]. According to different measurement planes, we evaluated CHD on the oblique sagittal plane and axial plane, respectively (Fig. 1). The coracoid overlap (CO) was defined as the distance between the glenoid and the tip of the coracoid process, which was measured on the axial plane [24] (Fig. 1). According to the previous study [29], the relative ratio of the coracoid length and humeral head diameter measured on the axial plane was defined as coracohumeral index (CHI) (Fig. 1). Shim et al. introduced two selected oblique sagittal planes (the en-face and Y-face) [31]. The en-face plane was the image in which the glenoid was the largest observed and the base of the coracoid process was in contact with the glenoid, and the Y-view was the first image medial to the glenoid where the scapular spine was in contact with the scapular body (Fig. 2). We evaluated subscapular muscle atrophy and fluid accumulation in these two planes (Fig. 2). In the en-face and Y-face planes, subscapular muscle atrophy was classified as grades I, II, and III according to the degree of atrophy. In en-face, the atrophy of SSC was evaluated according to the base-to-tip line (BTL) introduced by Shim et al. [31] In Y-face, the atrophy of SSC was graded based on the tangent line and its parallel line. A detailed description of classification in subscapular muscle atrophy was provided in Fig. 2. To further evaluate fluid accumulation on en-face and Y-face, we introduced a new index, namely fluid area ratio. According to the base-to-tip line (BTL), the fluid area ratio was defined as the ratio of the effusion area to the area surrounded by the coracoid process, glenoid, and BTL in the en-face (Fig. 2). To facilitate the measurement of the fluid area ratio (en-face) on MRI, this indicator was graded as ratio>0.5 and ratio<0.5.

The posterosuperior (supraspinatus, infraspinatus, teres minor) rotator cuff is anatomically adjacent to the SSC tendon. The retraction and severity of posterosuperior (PS) rotator cuff tears may affect the development and progression of SSC tendon lesions. To further investigate their relationship, the retraction of PS rotator cuff tears was classified according to the Patte classification (grade I ~ III) [31, 33]. We evaluated the severity of PS rotator cuff tear according to the thickness (full-thickness versus non full-thickness) and the number of tendon tears (normal/single versus multiple). Yoon et al. demonstrated that malposition (subluxation/dislocation) of the long head tendon of the biceps (LHB) on MRI was associated with a concurrent SSC full-thickness tear [26], and we evaluated the LHB malposition in the axial plane (Fig. 1). Turkmen et al. suggested that the prevalence of SSC tendon tears was higher in patients with subcoracoid cyst [33] (Fig. 1). In this study, the subcoracoid cyst was measured in the axial plane. Mostly, the greater tubercle cysts were considered to be associated with supraspinatus tendon tears [34, 35], clinicians speculated that the lesser tuberosity cysts (LTC) maybe be related to SSC tendon tears [27, 32]. We evaluated the presence of lesser tuberosity cysts and measured their maximum diameter in the fat-suppressed T2-weighted axial plane.

Clinical characteristics and indirect indicators were evaluated by the orthopaedists (LS and FW). We defined the diagnosis based on direct signs (changes in tendon morphology and signal on MRI) as MRI diagnosis, which was performed by the musculoskeletal radiologist and another orthopaedist (JCL). When disagreement persisted, a third reviewer (experienced shoulder surgeon: YNZ) was contacted to resolve the disagreement. All of the above investigators (LS, FW, JCL, YNZ, and musculoskeletal radiologist) were blinded to the arthroscopic diagnosis and grouping of the participant. The MRI diagnosis (direct signs) was also included as a candidate variable for evaluation and was classified into two types: considering SSC tendon tear or considering non-SSC tendon tear on MRI. All suspicious diagnoses based on direct signs were considered to be SSC tendon tears. Ultimately, eighteen candidate imaging indicators, including MRI diagnosis (direct signs), were assessed in the study along with 4 clinical characteristics.

### Statistical analysis

Categorical variables were described as the whole number, and continuous variables were expressed as means ± standard deviation. The significance of each variable in the training cohort was assessed by univariate logistic regression analysis firstly. Variables with a *p*-value < 0.05 were considered to be statistically significant in univariate logistic regression analysis. All variables at a significant level were further assessed in multivariate logistic regression (forward stepwise likelihood ratio method) to select the final independent risk factors. Variables with a *p*-value < 0.05 were considered to be statistically significant in multivariate logistic regression.

Continuous variables including age, CHD, CO, and CHI were directly analyzed in the logistic regression. Dichotomous variables such as gender, cause of injury, injured side, fluid accumulation, subcoracoid cyst, LHB dislocation/subluxation, and the number of PS rotator cuff tears were also directly analyzed in the logistic regression. For ordered categorical variables (atrophy, patte classification, and thickness of PS rotator cuff tear), we converted them into dichotomous variables with optimal scale regression before entering logistic regression.

According to the results of multivariate logistic regression, the “rms” package of R, version 4.0 (http://www.r-project.org/) was used to establish a nomogram. The nomogram was based on proportionally converting each regression coefficient in multivariate logistic regression to a 0- to 100-point scale. The total points were derived from the sum of the points for each independent variable and converted into predicted probability. The predictive performance of the nomogram is evaluated by concordance index (C index), decision curve analysis (DCA), and calibration with 1000 bootstrap samples. The performance of the predictive model was evaluated by the sensitivity, specificity, predictive values, and likelihood ratios.

## Results

### Evaluation and screening of predictors

A total of 660 patients met the inclusion criteria and the characteristics were presented in Table 1. In the training cohort, 184 patients entered the SSC tear group (Lafosse I/II: 138 patients; Lafosse III ~ V: 46 patients), and the remaining 276 patients were included in the non-SSC tear group. The mean age of both groups was 62.55 ± 9.03 and 59.76 ± 9.42 years, respectively. In the validation cohort, 86 patients entered the SSC tear group (Lafosse I/II: 65 patients; Lafosse III ~ V: 21 patients), and 114 patients were included in the non-SSC tear group. The mean age of these two groups was 63.12 ± 8.82 and 60.48 ± 9.13 years, respectively. The detailed information about these two cohorts was presented in Table 1.

After definition, conversion, and classification, univariate logistic regression was used to evaluate these 22 characteristics. The results of univariate logistic regression were presented in Table 1. Significant differences were found in 16 variables which were further evaluated by multivariate logistic regression. Variables for inclusion were carefully chosen, given the number of events available, to ensure parsimony of the final model. The forward stepwise likelihood ratio method was used in the multivariate logistic regression to identify the most related independent predictors of SSC tendon tears. All of the independent predictors were reported as odds ratios (95% CI). Eventually, CHD (oblique sagittal) (OR, 0.75; 95%CI, [0.67–0.84]), fluid accumulation (Y-face) (OR, 2.29; 95%CI, [1.20–4.38]), LHB dislocation/subluxation (OR, 3.62; 95%CI, [1.96–6.68]), number of PS rotator cuff tears (OR, 5.36; 95%CI, [3.12–9.22]), and MRI diagnosis (based on direct signs) (OR, 1.88; 95%CI, [1.06–3.32]) were highly associated with SSC tendon tears (p<0.05)(Table. 2).

**Table 2:** Result of multivariate logistic analysis

Variable	β	OR (95%CI)	*P* Value
CHD (oblique sagittal), mm	−0.29	0.75 (0.67–0.84)	<0.001
Fluid accumulation (Y-face)	0.83	2.29 (1.20–4.38)	0.012
LHB (dislocation/subluxation)	1.29	3.62 (1.96–6.68)	<0.001
Number of PS rotator cuff tears (≤1 or ≥ 2)	1.68	5.36 (3.12–9.22)	<0.001
MRI diagnosis (direct signs)	0.63	1.88 (1.06–3.32)	0.03

*CHD* Coracohumeral distance, *LHB* Long head of biceps tendon, *OR* Odds Ratio

### Nomogram and model performance

**Fig. 3:**
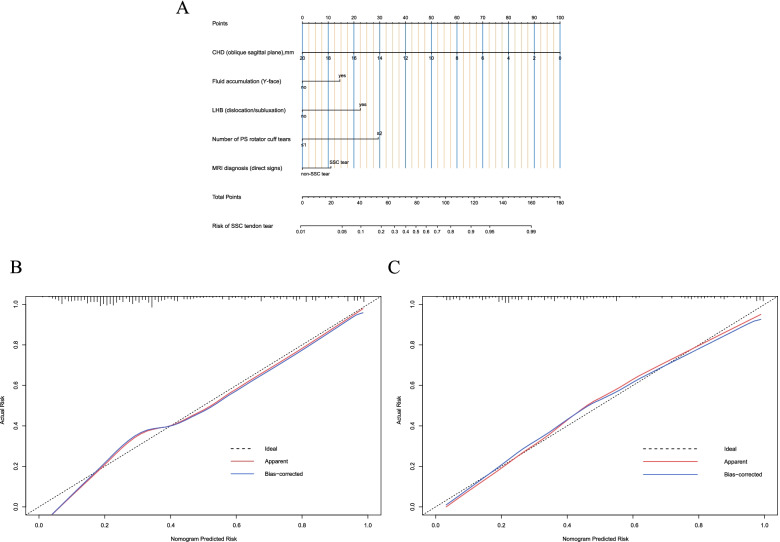
Nomogram and calibration. **A** Risk estimation nomogram on the SSC tendon tears. To use the nomogram, find the position of each variable on the corresponding axis, draw a line to the points axis for the number of points, add the points from all of the variables, and draw a line from the total points axis to determine the tear risk of SSC tendon at the lower line of the nomogram. e.g. If a patient was found to have 9 mm CHD, fluid accumulation in Y-face view, LHB dislocation, and multiple PS rotator cuff tears with suspected SSC tear on MRI, the total points of the nomogram was about 137. The corresponding risk estimation exceeds 95% which means the probability of a SSC tendon tear in this patient is over 95%. **B** Calibration plot (internal validation). Internal validation of the predictive performance of the nomogram in estimating the risk of SSC tendon tears with the 1000-sample bootstrapped calibration plot. The calibration curve demonstrated that our nomograms have good predictive performance. **C** External validation of the nomogram with another cohort of patients. The calibration curve demonstrated that the nomograms have good predictive performance in this cohort

A Nomogram to predict SSC tendon tears based on these 5 reliable predictors was shown in Fig. 3. The generated model was internally validated with the 1000 bootstrap validation method. This nomogram demonstrated good discriminative ability in estimating the risk of SSC tendon tears with a C index of 0.820 (0.782–0.858). In addition, calibration plots graphically showed good agreement on the risk estimation by the nomogram with 1000 resampling (Fig. 3). Patients with decreased CHD (oblique sagittal), LHB dislocation/subluxation, fluid accumulation (Y-face), multiple PS rotator cuff tears, and considering SSC tear on MRI (based on direct signs) were associated with higher nomogram points. Higher total points based on the sum of points for independent variables in the nomogram were associated with a greater risk of SSC tendon tears. In the validation cohort, the nomogram displayed a C index of 0.847 (95% CI, 0.794–0.900) for the risk estimation of SSC tendon tears with a good calibration curve for the risk estimation (Fig. 3). The Cohen’s Kappa values were calculated to assess intra- and inter-observer agreement reliability in nomogram (Kappa = 0.85 and 0.82 respectively).

**Fig. 4:**
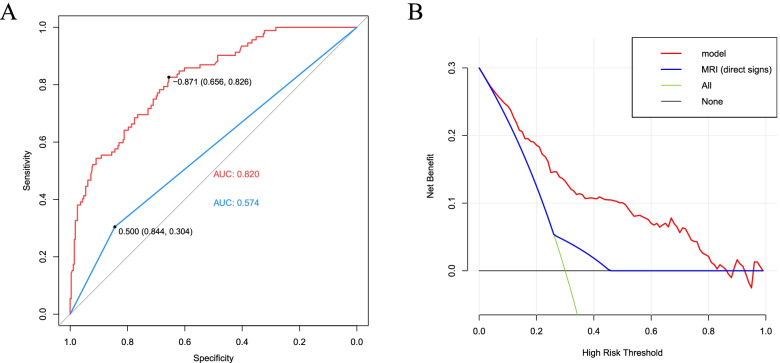
ROC and DCA curve. **A** AUC of two methods (nomogram: 0.820, MRI: 0.574). **B** DCA of two methods. DCA curve analysis showed this model (nomogram) has comparable clinical performance with higher clinical application value and better clinical practicability than MRI

We performed the receiver-operating characteristic (ROC) curve for this prediction model and MRI diagnosis based on direct signs (Fig. 4). DeLong test [36] was used to evaluate the statistical significance of AUCs (the area under the curve) between these two methods (0.820 vs 0.574). The result indicated that this nomogram had significantly higher accuracy than MRI diagnosis based on direct signs (*p* < 0.0001) for diagnosing SSC tendon tears. The diagnostic odds ratio (DOR) was used as a single measure of efficacy of a diagnostic test. A higher positive likelihood ratio meant a higher likelihood of diagnosing SSC tendon tear, while a smaller negative likelihood ratio meant a greater likelihood of ruling out SSC tendon tear. The result indicated that the risk prediction model achieved superior diagnostic performance in sensitivity (82.6% vs 30.4%), positive predictive value (61.5% vs 31.4%), negative predictive value (85% vs 78.5%), positive likelihood ratio (2.40 vs 1.95), negative likelihood ratio (0.27 vs 0.82) and diagnostic odds ratio (8.89 vs 2.38). Compared with diagnosis based on direct signs, the combination of these reliable predictors could yield better test discriminatory performance. The detailed outcomes were presented in Table 3. In addition, we also performed decision curve analysis (DCA) to assess the clinical utility of these two methods (Fig. 4). The result suggested that the prediction model can yield a good net benefit with better clinical utility than MRI diagnosis based on direct signs.

**Table 3:** Diagnostic performance of nomogram and MRI for predicting subscapularis tendon tears

Variable	Value-nomogram (95%CI)	Value-MRI (direct signs) (95%CI)
Area under ROC curve	0.820 (0.782–0.858)	0.574 (0.504–0.644)
Sensitivity, %	82.6 (76.2–87.6)	30.4 (21.5–41.0)
Specificity, %	65.6 (59.6–71.1)	84.4 (79.5–88.4)
Positive predictive value, %	61.5 (55.1–67.6)	39.4 (28.3–51.8)
Negative predictive value, %	85.0 (79.3–89.4)	78.5 (73.2–82.9)
Positive likelihood ratio	2.40 (2.01–2.86)	1.95 (1.29–2.95)
Negative likelihood ratio	0.27 (0.19–0.36)	0.82 (0.72–0.94)

*MRI* Magnetic resonance imaging, *ROC* Receiver operating characteristic, *CI* Confidence interval

According to the area under the ROC curve of this prediction model, we found that the best diagnostic sensitivity (82.6%) and specificity (65.6%) were obtained when the total nomogram score was 65, corresponding to a risk prediction value of 30%. Therefore, when the risk prediction value was > 30%, we defined it as the high-risk tear group, and when the risk prediction value was ≤30%, we defined it as the low-risk tear group.

## Discussion

Partial-thickness tear occurring in the upper 1/3 footprint was the main type of SSC tendon tear, which was difficult to identify the lesions according to direct signs (morphology and signal) on MRI. The combination of reliable predictors will help to comprehensively evaluate such injuries and improve diagnostic sensitivity. We first identified and screened out indicators with important predictive value in SSC tendon tears, and developed a nomogram model as an important tool to assist clinical diagnosis. The prediction model could provide the individualized risk prediction value. Whether in the training or validation cohort, the nomogram performed a good calibration curve for the risk estimation. An excellent intra- and inter-observer agreement based on the kappa consistency test has been achieved. These results showed that our prediction model had good repeatability and stability. According to the classification system of high and low-risk groups, doctors could identify individuals at high risk for SSC tendon tears, and give reasonable clinical recommendations to avoid omissions. Patients with decreased CHD (oblique sagittal), fluid accumulation (Y-face), LHB dislocation/subluxation, multiple (≥2) PS rotator cuff tears, and considering SSC tear on MRI (direct signs) were associated with a higher risk of SSC tendon tears. When evaluating SSC tendon injuries, clinicians should attach importance to the combined diagnostic value of these indirect predictors.

Previous studies implied that the morphology of the coracoid process could affect the development and progression of SSC tendinopathy [24, 29, 37]. Leite M. J et al. pointed out that coracoid distal length (CLD) and coracoid angle (CA) were associated with SSC tendon tears [38]. But in clinical practice, we found that it was difficult to select an ideal measurement plane on MRI according to the defined method, and the clinical practicability was poor. Based on previous literature reports, clinical importance, and practicality, three coracoid morphological parameters including CHD, CO, and CHI were considered potential candidates for SSC tendon tears [24, 29, 39]. To further evaluate the relationship between coracoid morphology and SSC tendon tears, we measured CHD (axial plane), CHD (oblique sagittal plane), CHI (axial plane), and CO (axial plane) respectively. The result indicated that a decreased CHD (oblique sagittal plane) was highly associated with SSC tendon tears, which had greater predictive value and diagnostic sensitivity than the other three indicators. The smaller CHD, the higher risk of subscapular tendon tear.

Anatomically, the SSC tendon is interdependent with the PS rotator cuff and LHB to support the biceps pully structure. Some scholars suggested that the lesion of PS rotator cuff and LHB were associated with SSC tendon tears [25, 26, 40]. In our study, the result of multivariate logistic regression indicated that LHB subluxations/dislocation and multiple PS rotator cuff tears were significantly associated with SSC tendon tears. Patients with these risk factors were prone to acquiring SSC tendon tears.

Previous studies attempted to reveal the relationship between fluid signal and SSC tendon tears [27, 31, 33]. They suggested that patients with subcoracoid cyst, lesser tuberosity cyst, and fluid accumulation around the SSC were more likely to have SSC tendon tears. In this study, we introduced a new index, namely the fluid area ratio. To facilitate the evaluation of this indicator on MRI we divided the fluid area ratio into two-level, namely ratio>0.5 and ratio<0.5. Although we did not find lesser tuberosity cyst, fluid area ratio, and subcoracoid cyst were associated with SSC tendon tears, the result recommended fluid accumulation (Y-face) as a strong predictor of SSC tendon tears. Patients with fluid accumulation (Y-face) on MRI indicated a higher risk of SSC tendon tears.

Eventually, CHD (oblique sagittal), fluid accumulation (Y-face), LHB dislocation/subluxation, the number of PS rotator cuff tears, and MRI diagnosis (based on direct signs) were determined as critical predictors associated with SSC tendon tears. The current study provided new insight into evaluating SSC tendon tears. Compared with the traditional diagnosis based on the direct signs of the lesions, the combination of the above reliable predictors can comprehensively evaluate the SSC tendon injury on MRI with better diagnostic performance. The classification of high and low-risk groups can assist doctors in identifying patients at high risk for SSC tendon tears and make reasonable clinical recommendations.

As a new prediction tool, this nomogram was convenient to reach a consensus on risk assessment to assist clinicians in diagnosis. But there were still some limitations in our study. First, this analysis was based on the data of a single institution, it is necessary to validate the results from other centers in the future. In addition, a recent systematic review and meta-analysis found that even the most sensitive clinical test for evaluating SSC tendon tears had a sensitivity of only about 50% [41]. Although clinical tests are routine procedures, we did not include them in this study because of incomplete clinical data and the limited accuracy reported in the previous literature [42]. No single clinical test was sufficiently reliable to diagnose SSC tendon tears, a comprehensive analysis combing the prediction model, MRI diagnosis, and no less than two clinical tests was recommended.

## Conclusions

This research provided a novel prediction model of SSC tendon tears which could assist clinicians in diagnosis. When evaluating the severity of SSC tendon injuries, we should give increasing attention to the combined diagnostic value of these sensitive predictors. This model provided satisfactory prediction performance which is convenient for clinicians to identify patients at high risk for SSC tendon tears to avoid omission.

## Data Availability

The datasets used and/or analysed during the current study are available from the corresponding author on reasonable request.

## Abbreviations

CHD: Coracohumeral distance
CO: Coracoid overlap
CHI: Coracohumeral index
ROC: Receiver-operating characteristic
DCA: Decision curve analysis
LHB: Long head of the biceps
LTC: Lesser tuberosity cyst
SSC: Subscapularis
MRI: Magnetic resonance imaging
OR: Odds ration
PS: Posterosuperior
MRA: Magnetic resonance arthrography
DOR: Diagnostic odds ratio

## References

[1] Mitterer M, Matis N, Steiner G, Vasvary I, Ortmaier R (2021). Muscle volume imbalance may be associated with static posterior humeral head subluxation. BMC Musculoskelet Disord.

[2] Waldt S, Bruegel M, Mueller D (2007). Rotator cuff tears: assessment with MR arthrography in 275 patients with arthroscopic correlation. Eur Radiol.

[3] Lee JH, Yoon YC, Jee S, Kwon JW, Cha JG, Yoo JC (2014). Comparison of three-dimensional isotropic and two-dimensional conventional indirect MR arthrography for the diagnosis of rotator cuff tears. Korean J Radiol.

[4] Narasimhan R, Shamse K, Nash C, Dhingra D, Kennedy S (2016). Prevalence of SSC tears and accuracy of shoulder ultrasound in pre-operative diagnosis. Int Orthop.

[5] Lin L, Yan H, Xiao J (2016). The diagnostic value of magnetic resonance imaging for different types of SSC lesions. Knee Surg Sports Traumatol Arthrosc.

[6] Sela Y, Eshed I, Shapira S (2015). Rotator cuff tears: correlation between geometric tear patterns on MRI and arthroscopy and pre- and postoperative clinical findings. Acta Radiol.

[7] Ward JRN, Lotfi N, Dias RG, McBride TJ (2018). Diagnostic difficulties in the radiological assessment of SSC tears. J Orthop.

[8] Furukawa R, Morihara T, Arai Y (2014). Diagnostic accuracy of magnetic resonance imaging for SSC tendon tears using radial-slice magnetic resonance images. J Shoulder Elb Surg.

[9] Matsushita R, Yokoya S, Negi H, Matsubara N, Akiyama Y, Adachi N (2022). Evaluation of SSC tendon tears of the anterosuperior aspect using radial-sequence magnetic resonance imaging. JSES Int.

[10] Malavolta EA, Assunção JH, Gracitelli MEC, Yen TK, Bordalo-Rodrigues M, Ferreira Neto AA (2019). Accuracy of magnetic resonance imaging (MRI) for SSC tear: a systematic review and meta-analysis of diagnostic studies. Arch Orthop Trauma Surg.

[11] Naimark M, Zhang AL, Leon I, Trivellas A, Feeley BT, Ma CB (2016). Clinical, radiographic, and surgical presentation of SSC tendon tears: a retrospective analysis of 139 patients. Arthroscopy.

[12] Banerjee M, Müller-Hübenthal J, Grimme S (2016). Moderate value of non-contrast magnetic resonance imaging after non-dislocating shoulder trauma. Knee Surg Sports Traumatol Arthrosc.

[13] Adams CR, Schoolfield JD, Burkhart SS (2010). Accuracy of preoperative magnetic resonance imaging in predicting a SSC tendon tear based on arthroscopy. Arthroscopy.

[14] Yoo JC, Rhee YG, Shin SJ (2015). SSC tendon tear classification based on 3-dimensional anatomic footprint: a cadaveric and prospective clinical observational study. Arthroscopy.

[15] Cigolotti A, Biz C, Lerjefors E, de Iudicibus G, Belluzzi E, Ruggieri P (2021). Medium- to long-term clinical and functional outcomes of isolated and combined SSC tears repaired arthroscopically. Arch Med Sci.

[16] Liu Y, Lafosse L, Opsomer G, Villain B, Kempf JF, Collin P (2020). Ten-year clinical and magnetic resonance imaging evaluation after repair of isolated SSC tears. JSES Int.

[17] Nové-Josserand L, Hardy MB, Leandro Nunes Ogassawara R, Carrillon Y, Godenèche A (2012). Clinical and structural results of arthroscopic repair of isolated SSC tear. J Bone Joint Surg Am.

[18] Hasler A, Ker A, Passon T, Tondelli T, Gerber C, Wieser K (2022). Nonoperatively managed small- to medium-sized SSC tendon tears: magnetic resonance imaging evaluation with a minimum of 5 years of follow-up. JSES Int..

[19] Yoon TH, Kim SJ, Choi YR, Keum HS, Chun YM (2021). Clinical outcomes for isolated SSC tears with advanced fatty infiltration: nonoperative treatment versus arthroscopic single-row repair. Orthop J Sports Med..

[20] Yoon TH, Kim SJ, Choi YR, Cho JT, Chun YM (2021). Arthroscopic revision rotator cuff repair: the role of previously neglected SSC tears. Am J Sports Med.

[21] Lee SH, Nam DJ, Kim SJ, Kim JW (2017). Comparison of clinical and structural outcomes by SSC tendon status in massive rotator cuff tear. Am J Sports Med.

[22] Park JY, Chung SW, Lee SJ (2016). Combined SSC tears in massive Posterosuperior rotator cuff tears: do they affect postoperative shoulder function and rotator cuff integrity?. Am J Sports Med.

[23] Ramadan LB, Baptista E, Souza FF (2020). Diagnostic accuracy of preoperative magnetic resonance imaging for detecting SSC tendon tears: a diagnostic test study. Sao Paulo Med J.

[24] Leite MJ, Sá MC, Lopes MJ, Matos RM, Sousa AN, Torres JM (2019). Coracohumeral distance and coracoid overlap as predictors of SSC and long head of the biceps injuries. J Shoulder Elb Surg.

[25] Mehta SK, Teefey SA, Middleton W, Steger-May K, Sefko JA, Keener JD (2020). Prevalence and risk factors for development of SSC and biceps pathology in shoulders with degenerative rotator cuff disease: a prospective cohort evaluation. J Shoulder Elb Surg.

[26] Yoon JS, Kim SJ, Choi YR, Lee W, Kim SH, Chun YM (2018). Medial subluxation or dislocation of the biceps on magnetic resonance arthrography is reliably correlated with concurrent SSC full-thickness tears confirmed arthroscopically. Biomed Res Int.

[27] Cetinkaya M, Öner AY, Ataoglu MB, Ozer M, Ayanoglu T, Kanatli U (2017). Lesser tuberosity cysts and their relationship with SSC tears and subcoracoid impingement. J Orthop Sci.

[28] Lafosse L, Jost B, Reiland Y, Audebert S, Toussaint B, Gobezie R (2007). Structural integrity and clinical outcomes after arthroscopic repair of isolated SSC tears. J Bone Joint Surg Am.

[29] Zhang H, Zhang Q, Li ZL (2019). Coracohumeral index and coracoglenoid inclination as predictors for different types of degenerative SSC tendon tears. Int Orthop.

[30] Meyer DC, Zimmermann SM, Wieser K, Bensler S, Gerber C, Germann M (2016). Lengthening of the SSC tendon as a sign of partial tearing in continuity. J Shoulder Elb Surg.

[31] Shim JW, Pang CH, Min SK, Jeong JY, Yoo JC (2019). A novel diagnostic method to predict SSC tendon tear with sagittal oblique view magnetic resonance imaging. Knee Surg Sports Traumatol Arthrosc.

[32] Wissman RD, Ingalls J, Hendry D, Gorman D, Kenter K (2012). Cysts within and adjacent to the lesser tuberosity: correlation with shoulder arthroscopy. Skelet Radiol.

[33] Turkmen I, Altun G, Celik H, Bilsel K (2020). Can subcoracoid cyst formation be a sign of anterosuperior rotator cuff tears and biceps pulley lesions? A prospective radiologic and arthroscopic correlation study. J Shoulder Elb Surg.

[34] Chillemi C, Paglialunga C, Guerrisi M, Mantovani M, Osimani M (2020). Arthroscopic Transosseous repair of rotator cuff tear and greater tuberosity cysts. Arthrosc Sports Med Rehabil.

[35] Chin K, Chowdhury A, Leivadiotou D, Marmery H, Ahrens PM (2019). The accuracy of plain radiographs in diagnosing degenerate rotator cuff disease. Shoulder Elbow.

[36] DeLong ER, DeLong DM, Clarke-Pearson DL (1988). Comparing the areas under two or more correlated receiver operating characteristic curves: a nonparametric approach. Biometrics.

[37] Hodax JD, Shah KN, Campbell SE, Cameron KL, Owens BD (2021). Measurement of the coracohumeral distance on magnetic resonance imaging in a large patient cohort. J Shoulder Elb Surg.

[38] Leite MJ, Pinho AR, Sá MC, Silva MR, Sousa AN, Torres JM (2020). Coracoid morphology and humeral version as risk factors for SSC tears. J Shoulder Elb Surg.

[39] Zhu S, Tan J, Wu D, Hu N, Huang W, Chen H (2021). Bilateral coracohumeral distance discrepancy is associated with SSC tear in rotator cuff rupture patients. Knee Surg Sports Traumatol Arthrosc..

[40] Kim BR, Lee J, Ahn JM (2021). Predicting the clinically significant SSC tendon tear: malposition and tear of the long head of the biceps tendon on shoulder magnetic resonance imaging. Acta Radiol..

[41] Lädermann A, Collin P, Zbinden O, Meynard T, Saffarini M, Chiu JC (2021). Diagnostic accuracy of clinical tests for SSC tears: a systematic review and Meta-analysis. Orthop J Sports Med.

[42] Gismervik S, Drogset JO, Granviken F, Rø M, Leivseth G (2017). Physical examination tests of the shoulder: a systematic review and meta-analysis of diagnostic test performance. BMC Musculoskelet Disord.

